# Dissociation of the Hepatic and Pulmonary Axes in Alpha-1 Antitrypsin Deficiency: Independent Trajectories of Organ-Specific Disease

**DOI:** 10.3390/biom16070940

**Published:** 2026-06-24

**Authors:** Juan Luis Rodríguez Hermosa, Soha Esmaili, Iman Esmaili, Maria Torres-Duran, Hanan Tanash, Alice M. Turner, Carlota Rodríguez-García, Miriam Barrecheguren, Jens-Ulrik Stæhr Jensen, Vincent Bunel, Angelo Guido Corsico, Kenneth R. Chapman, Jean-François Mornex, Eva Bartošovská-Klinková, Beatriz Lara, José Luis López-Campos, Christian F. Clarenbach, Emily F. A. van ’t Wout, Mariano Fernandez-Acquier, Myriam Calle Rubio

**Affiliations:** 1 Pulmonology Department, Hospital Clínico San Carlos, 28040 Madrid, Spain; jlrodr01@ucm.es; 2 Instituto de Investigación Sanitaria del Hospital Clínico San Carlos (IdISSC), 28040 Madrid, Spain; soha@esmaili.ws; 3 Department of Medicine, School of Medicine, Universidad Antonio Nebrija, 28248 Madrid, Spain; 4 Pulmonology Department, Hospital Universitario La Zarzuela and Hospital Quirónsalud San Jose, 28023 Madrid, Spain; 5 ISNS Data Analytics and Research, Vancouver, BC V6Z 1Y6, Canada; 6 NeumoVigo I+i Research Group, Pneumology Department, Hospital Álvaro Cunqueiro, IIS Galicia Sur, 36211 Vigo, Spain; mtordur@gmail.com; 7 Centro de Investigación Biomédica en Red de Enfermedades Respiratorias (CIBERES), Instituto de Salud Carlos III, 28029 Madrid, Spain; miriam.barrecheguren@vallhebron.cat (M.B.); lcampos@separ.es (J.L.L.-C.); 8 Department of Respiratory Medicine and Allergology, Skåne University Hospital, Lund University, 214 28 Malmö, Sweden; hanan.tanash@med.lu.se; 9 Respiratory Medicine, University Hospitals Birmingham NHS Foundation Trust, Birmingham B15 2GW, UK; a.m.turner@bham.ac.uk; 10 Pneumology Department, Complejo Hospitalario Universitario de Ferrol, 15405 El Ferrol, Spain; crodgar5@gmail.com; 11 Department of Pneumology, Universitary Hospital Vall d’Hebron/Vall d’Hebron Institut de Recerca (VHIR), Vall d’Hebron Barcelona Hospital Campus, and European Reference Network on Rare Respiratory Diseases (ERN LUNG), 08035 Barcelona, Spain; 12 Section of Respiratory Medicine, Department of Medicine, Herlev and Gentofte Hospital, University of Copenhagen, 2830 Gentofte, Denmark; jens.ulrik.jensen@regionh.dk; 13 Department of Pulmonology, Allergology, and Transplantation, AP-HP, Bichat Hospital, Paris Cité University, Inserm, Centre de Recherche de l’Inflammation, F-75018 Paris, France; vincent.bunel@gmail.com; 14 Department of Internal Medicine and Therapeutics, University of Pavia, 27100, Pavia, Italy; a.corsico@smatteo.pv.it; 15 Division of Respiratory Medicine, Department of Medicine, University of Toronto, Toronto, ON M5T 2S8, Canada; ken.chapman.airways@gmail.com; 16 Hospices Civils de Lyon, OrphaLung, Respifil and CHU de Saint Etienne, F-69000 Lyon, France; jean-francois.mornex@univ-lyon1.fr; 17 Department of Pneumology, Thomayer Hospital, First Faculty of Medicine, Charles University, CZ-121 08 Prague, Czech Republic; eva.bartosovska@ftn.cz; 18 Department of Respiratory Medicine, University Hospitals Coventry and Warwickshire NHS Trust, Coventry CV2 2DX, UK; beatriz.lara@uhcw.nhs.uk; 19 Unidad Médico-Quirúrgica de Enfermedades Respiratorias, Instituto de Biomedicina de Sevilla (IBiS), Hospital Universitario Virgen del Rocío, Universidad de Sevilla, 41013 Sevilla, Spain; 20 Pulmonary Clinic, University Hospital Zurich, University of Zurich, 8091 Zurich, Switzerland; christian.clarenbach@usz.ch; 21 Department of Pulmonology, Leiden University Medical Centre, 2333 ZA Leiden, The Netherlands; e.f.a.van_t_wout@lumc.nl; 22 Hospital Zonal Especializado de Agudos y Crónicos Dr. Antonio Cetrángolo, Vicente López, Buenos Aires 1802, Argentina; acquier@hotmail.com

**Keywords:** alpha-1 antitrypsin deficiency, liver fibrosis, pulmonary emphysema, phenotypic dominance, geometric mapping, risk stratification, *SERPINA1*

## Abstract

The interindividual phenotypic heterogeneity in Alpha-1 Antitrypsin Deficiency (AATD), despite a shared genetic etiology (the Z-allele of *SERPINA1*), is explained by the interaction of dual pathogenic mechanisms (gain-of-function vs. loss-of-function), additional genetic modifiers, and environmental or metabolic factors. Building on recent evidence suggesting divergent disease trajectories, we investigated whether pulmonary and hepatic impairments represent coupled manifestations or independent clinical dimensions within a large European cohort. Methods: This international multicenter study utilized the European Alpha-1 Research Collaboration (EARCO) registry (*n* = 1217). Pulmonary and hepatic severities were quantified using concurrent 0.0–10.0 composite indices. Independence was evaluated via partial Spearman correlations, multivariable multinomial regression, and geometric mapping across a continuous phenotypic space. Results: Cross-domain correlations between respiratory metrics and liver stiffness were near zero (r = −0.03), demonstrating statistical independence. Phenotypic dominance classification isolated distinct profiles; the lung-dominant group exhibited a higher age (57.0 vs. 54.0 years; *p* < 0.001) and tobacco exposure, while the liver-dominant group registered a higher body mass index (25.8 vs. 24.4 kg/m^2^; *p* < 0.001). Multivariable models identified age (OR 1.03; 95% CI 1.02–1.05) and smoking as independent predictors of lung dominance, whereas body mass index was independently associated with liver dominance (OR 1.04; 95% CI 1.01–1.07). Geometric mapping revealed advanced disease clusters at orthogonal margins rather than forming a systemic continuum. Conclusions: Hepatic and pulmonary impairments in AATD operate as independent clinical dimensions modulated by distinct metabolic and environmental factors. Risk stratification must transition toward organ-specific prognostic models.

## 1. Introduction

Alpha-1 antitrypsin deficiency (AATD) is a monogenic disorder characterized by an increased susceptibility to early-onset pulmonary emphysema and progressive hepatic fibrosis [1,2]. The most common mutation leading to severe AATD is the *SERPINA1* Z variant, corresponding to the Glu342Lys missense variant and registered in genetic databases as rs28929474. The pathophysiological mechanisms underlying end-organ damage in homozygous (ZZ) individuals diverge intrinsically: respiratory impairment is primarily driven by a loss-of-function protease–antiprotease imbalance, whereas liver disease stems from a toxic gain-of-function mechanism secondary to the intrahepatocytic accumulation of polymerized mutant protein [3,4]. These different mechanisms may explain why the organs are affected at different times. Longitudinal screening data indicate that pulmonary symptoms typically manifest around the fourth decade of life, particularly in smokers, whereas never-smokers often maintain preserved lung function [5]. Conversely, adult-onset liver disease usually appears in the fourth or fifth decade of life [6]. Approximately one-third of adults with the ZZ genotype develop significant liver fibrosis. Notably, an observational study found that patients with lung disease had a significantly longer time from AATD diagnosis to liver disease diagnosis (2.2 vs. 0.2 years) compared with those without lung disease [7]. Despite a shared genetic etiology, predominantly defined by the *SERPINA1* Z variant, the clinical presentation of AATD exhibits marked inter-individual heterogeneity [8]. This variable penetrance indicates that genetic burden alone is insufficient to predict the specific trajectory or magnitude of organ-specific damage [9]. Furthermore, organ-specific risk factors have been identified: pulmonary disease progression is strongly influenced by smoking and environmental exposures, while liver disease progression is associated with metabolic syndrome and alcohol consumption [10].

Current management guidelines recognize this distinction and recommend the independent assessment of both organs, with monitoring of liver involvement primarily for the ZZ and SZ genotypes, alongside specific considerations for those with the MZ genotype [11,12]. Providing greater insight into the relationship between liver and lung involvement is essential, as decoupled progression requires organ-specific risk stratification models.

Therefore, the main objective of this study was to analyze hepatic and pulmonary dissociation in the EARCO cohort, define categories of clinical dominance based on objective severity indices, and identify the independent clinical determinants driving organ-specific disease trajectories.

## 2. Materials and Methods

### 2.1. Study Design and Setting

This international, multicenter, cross-sectional study utilized baseline data systematically collected from the European Alpha-1 Research Collaboration (EARCO) clinical registry [13]. EARCO is a clinical research collaboration of the European Respiratory Society (ERS) that aims to answer fundamental questions about the epidemiology, genetics, pathophysiology, clinical treatment, and prognosis of AATD-related lung diseases. Individuals diagnosed with AATD, AAT serum levels < 11 µM (57 mg/dL), and/or deficiency defined as heterozygous or homozygous combinations of proteinase inhibitor genotypes ZZ, SZ, and other rare functionally deficient variants were eligible to participate. For this specific analysis, the observational framework captured comprehensive, concurrent multi-organ clinical data at the point of registry enrollment, establishing a robust cross-sectional baseline to evaluate the structural and functional architecture of hepatic and pulmonary disease [14].

### 2.2. Study Population and Eligibility Criteria

The source population comprised adults with genetically confirmed AATD enrolled in the EARCO registry. For this study, participants registered in EARCO between February 2020 and March 2025 were included. Out of a total of 3879 patients with AATD, 1217 were identified as having concurrent baseline pulmonary and hepatic evaluations. To ensure internal validity and prevent artifactual bias in cross-organ phenotyping, eligibility for the primary analytic cohort was strictly restricted to these individuals. Specifically, inclusion required complete data for the clinical variables needed to compute both the Liver Involvement Score (LIS) and the Pulmonary Involvement Score (PIS). These mandatory variables included concurrent pulmonary metrics (forced expiratory volume in one second [FEV1], forced vital capacity [FVC], and diffusing capacity of the lungs for carbon monoxide [DLCO]) and hepatic metrics (liver stiffness measurement [LSM], alanine aminotransferase [ALT], aspartate aminotransferase [AST], and platelet count for computation of the FIB-4 index). A strict complete-case analysis framework was implemented to avoid the introduction of imputation-related covariance structures that could artificially distort cross-domain associations. To assess potential selection bias, baseline characteristics of the included study cohort were compared with those of patients excluded due to missing dominance scores (Supplementary Table S1).

### 2.3. Clinical Measurements and Temporal Framework

Standardized clinical assessments captured demographics (age, biological sex, body mass index [BMI]), environmental exposures (smoking status, categorized alcohol consumption), and relevant baseline comorbidities. To quantify metabolic risk, a composite metabolic burden (MFB) score was constructed from three primary components: BMI, clinical diabetes (stratified by the presence or absence of macro/microvascular complications), and hypertension. Each component was standardized (z-score), summed, and re-standardized to generate a continuous metabolic burden scale. For categorical analyses, patients were stratified into three tiers (low, intermediate, and high MFB) based on the statistical tertiles of the distribution. Genetic burden was defined by the *SERPINA1* Z-allele count: 0 (non-Z genotypes), 1 (heterozygous), or 2 alleles (homozygous ZZ). Pulmonary evaluation included forced expiratory volume in one second (FEV1), forced vital capacity (FVC), and diffusing capacity of the lungs for carbon monoxide (DLCO), all expressed as percentages of predicted values according to the Global Lung Function Initiative (GLI) reference equations [15]. Hepatic phenotyping required transient elastography to record liver stiffness measurement (LSM) and controlled attenuation parameter (CAP), alongside concurrent biochemical evaluation—specifically serum alanine aminotransferase, aspartate aminotransferase, and platelet count—to compute the Fibrosis-4 (FIB-4) index [16]. All clinical, functional, and biochemical measurements were extracted strictly from the baseline registry visit, ensuring a concurrent temporal window for orthogonal cross-organ phenotyping.

### 2.4. Composite Indices and Phenotypic Classification

Organ-specific disease burdens were quantified using two parallel 0.0–10.0 continuous severity scales to capture multidimensional structural and functional damage that isolated metrics (e.g., FEV1 or LSM) cannot fully represent. The LIS is a composite index integrating structural, biochemical, and clinical hepatic parameters. Specifically, the 10.0-point scale assigns 0.0 to 5.0 points derived proportionally from LSM (capped at values ≥ 9.5 kilopascals), 0.0 to 3.0 points derived proportionally from the FIB-4 index (a validated composite of age, aspartate aminotransferase, alanine aminotransferase, and platelet count, capped at values ≥ 3.25), and 2.0 discrete points for the documented clinical presence of cirrhosis or hepatocellular carcinoma. The PIS is a complementary 0.0–10.0 continuous metric representing cumulative pulmonary structural and functional impairment. It was constructed as a domain-based index incorporating airflow obstruction (FEV1/FVC), FEV1 impairment, DLCO impairment, and structural lung damage. Domains were scaled continuously across predefined severity ranges, and the total observed score was prorated to a 0–10 scale according to the maximum possible score among the available domains. Detailed mathematical formulas for both PIS and LIS are provided in the Supplementary Methods.

To operationalize divergent disease trajectories, phenotypic dominance was classified using the standardized difference between these composite indices, calculated as Z(LIS) − Z(PIS). Patients were stratified into three mutually exclusive categories: liver-dominant if the standardized difference was strictly >+1.0 standard deviations (SD), lung-dominant if the difference was < −1.0 SD, and mixed/intermediate if the score fell between −1.0 and +1.0 SD. This ±1.0 SD threshold was selected a priori as a distribution-based criterion to identify pronounced organ imbalance while preserving an adequately sized intermediate reference group. To evaluate the robustness of this classification, sensitivity analyses utilizing alternative threshold definitions (±0.5 SD and ±1.5 SD) were also performed (Supplementary Tables S2 and S3).

### 2.5. Statistical Analysis and Geometric Mapping

Descriptive statistics utilized medians with interquartile ranges for continuous non-parametric variables and as absolute frequencies with proportions for categorical data. Between-group baseline differences across the three phenotypic dominance categories were evaluated using the Kruskal–Wallis test for continuous variables and chi-square or Fisher’s exact tests for categorical variables, as appropriate. For complementary analyses assessing the association between selected clinical/genetic strata and continuous organ-specific outcomes, analysis of variance or general linear models were used; F-statistics and η^2^ effect sizes were reported to quantify the magnitude of between-group differences. Cross-domain biomarker independence was quantified using partial Spearman rank correlation coefficients to accommodate non-linear monotonic relationships, while simultaneously adjusting for age, biological sex, and BMI to prevent confounding by shared demographic and metabolic drivers.

To map the continuous hepatic and pulmonary phenotypic space, normalized LIS and PIS values underwent geometric transformation into a ternary coordinate system, projecting patient distributions across axes of relative pulmonary burden, relative hepatic burden, and low combined organ burden (resilience). Multivariable multinomial logistic regression was subsequently employed to identify independent clinical and genetic predictors of phenotypic dominance, utilizing the mixed/intermediate phenotype as the reference outcome. The specified model simultaneously adjusted for age, BMI, sex, smoking status, alcohol exposure, and Z-allele count. Prior to modeling, multicollinearity was assessed using variance inflation factors, confirming the absence of problematic collinearity (Supplementary Table S4). Multivariable model diagnostics and sensitivity analyses across alternative phenotypic definitions confirmed the high stability of the observed associations (Supplementary Table S3). Additionally, to reduce reliance on categorical thresholds, complementary continuous analyses were performed using restricted cubic spline models to evaluate linear and non-linear associations between LIS and PIS across the full severity range (Supplementary Tables S5A and S5B). The continuous dominance score, defined as Z(LIS) − Z(PIS), was also modeled using restricted cubic splines as an additional threshold-free sensitivity analysis (Supplementary Table S5C).

### 2.6. Ethical Considerations

The study was conducted in strict accordance with the ethical standards established by the Declaration of Helsinki. The EARCO registry protocol was registered at www.clinicaltrials.gov (ID: NCT04180319, registration date posted on 27 November 2019), and is hosted at www.earco.org. The study protocol received central ethical approval from the Research Ethics Committee of the Vall d’Hebron University Hospital, Barcelona, Spain (PR(AG)480/2018), and was subsequently approved by all participating centers. Written informed consent was obtained from all participants. This study was endorsed by the Core Network AATD of the European Reference Network LUNG (ERN-LUNG).

## 3. Results

### 3.1. Clinical Characteristics Across Phenotypic Dominance Categories

Baseline demographic, genetic, and clinical parameters were evaluated to establish the foundational profile of the study cohort. Patients were stratified into three mutually exclusive phenotypic dominance categories to contrast the independent distribution of organ-specific disease burden.

Table 1 presents the baseline demographic, exposure, and clinical characteristics of the cohort, stratified by phenotypic dominance category. While primary stratification by classic *SERPINA1* genotype burden demonstrated the expected overall gradients of disease severity (Table S1), classification by phenotypic dominance isolated distinct organ-specific profiles. The lung-dominant group recorded the highest median age (57.0 years) and the largest proportion of ex-smokers (67.5%), corresponding to reduced pulmonary function metrics, including a median FEV1 of 48.0% predicted and an FEV1/FVC ratio of 0.44. In contrast, the liver-dominant group registered a higher median BMI (25.8 kg/m^2^) and a greater proportion of never-smokers (57.1%). Patients in this category maintained preserved pulmonary function (median FEV1 95.0% predicted) alongside elevated structural and biochemical hepatic markers, with a median liver stiffness of 6.4 kPa and a median FIB-4 index of 1.30. Individuals homozygous for the Z-allele (Z-count = 2) constituted the majority in both the lung-dominant (81.6%) and liver-dominant (64.1%) categories. Hepatic steatosis, quantified by the controlled attenuation parameter (CAP), exhibited no statistically significant variation across the three phenotypic groups (*p* = 0.115). Exploratory categorical analyses using clinical obesity (BMI ≥ 30 kg/m^2^) as an available marker of metabolic burden demonstrated a significantly higher prevalence of obesity among liver-dominant participants (132/466, 28.3%) than among lung-dominant participants (90/475, 18.9%) (OR = 1.69, 95% CI 1.22–2.33, *p* = 0.001). Furthermore, the proportion of liver-dominant phenotypes increased progressively across ascending BMI categories (Supplementary Figure S1).

### 3.2. Multi-Biomarker Dissociation of the Hepatic and Pulmonary Axes

The statistical association between pulmonary and hepatic biomarkers was evaluated through partial correlation analysis and direct variable visualization to quantify the independence of organ-specific disease trajectories.

**Figure 1:**
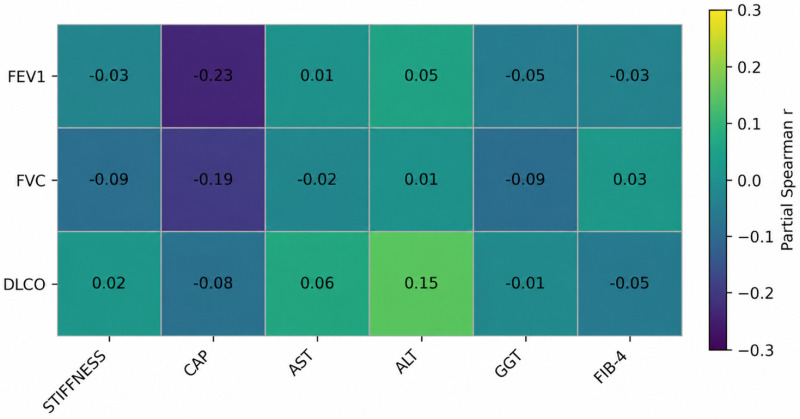
Cross-Domain Hepatic and Pulmonary Association Heatmap. Note. Heatmap displaying partial Spearman correlation coefficients (r) between pulmonary variables (FEV1, forced expiratory volume in one second; FVC, forced vital capacity; DLCO, diffusing capacity of the lungs for carbon monoxide) and hepatic variables (liver stiffness; CAP, controlled attenuation parameter; AST, alanine aminotransferase; ALT, aspartate aminotransferase; GGT, gamma-glutamyl transferase; FIB-4, Fibrosis-4 index). Correlations are adjusted for age, male sex, and body mass index (BMI). The color scale ranges from −0.30 to 0.30, where cooler colors (purples) indicate inverse associations and warmer colors (greens) indicate positive associations. Partial Spearman methods were applied directly to avoid residualization bias.

Figure 1 illustrates the adjusted cross-domain associations between pulmonary function and hepatic clinical markers. Partial Spearman correlation coefficients between primary pulmonary metrics and liver stiffness were near zero, with r values of −0.03 for FEV1, −0.09 for FVC, and 0.02 for DLCO. Associations between pulmonary measures and hepatic biochemical markers, including AST, ALT, GGT, and the FIB-4 index, ranged from −0.09 to 0.15. The largest observed cross-domain correlations were inverse associations between pulmonary function and hepatic steatosis (CAP), specifically r = −0.23 for FEV1 and r = −0.19 for FVC. All cross-organ correlation coefficients remained below an absolute magnitude of 0.25, demonstrating weak statistical coupling between clinical markers of the hepatic and pulmonary domains.

**Figure 2:**
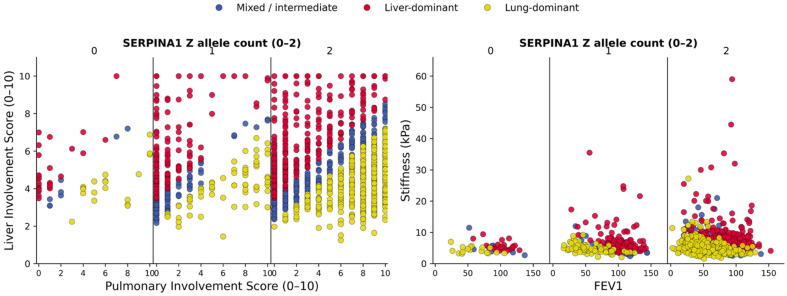
Genotype-Stratified Direct-Variable Validation of Phenotypic Dominance. Note. Faceted scatter plots illustrating the distribution of disease severity across phenotypic dominance categories, stratified by *SERPINA1* Z-allele count (0, 1, and 2). The left plot displays the relationship between the Pulmonary Involvement Score (PIS, range 0–10) and the Liver Involvement Score (LIS, range 0–10). The right plot displays the relationship between raw clinical variables: forced expiratory volume in one second (FEV1, % predicted) on the x-axis and liver stiffness (kPa) on the y-axis. Data points are colored by phenotypic dominance category: mixed/intermediate (blue), liver-dominant (red), and lung-dominant (yellow). Phenotypic dominance categories were defined using the standardized difference between the Liver Involvement Score and the Pulmonary Involvement Score, calculated as Z(LIS) − Z(PIS). Patients were classified as liver-dominant if the score was >+1.0 standard deviation (SD), lung-dominant if the score was <−1.0 SD, and mixed/intermediate if the score fell between −1.0 and +1.0 SD. Abbreviations: SD, standard deviation.

Figure 2 presents the distribution of patients across hepatic and pulmonary severity axes using composite indices and direct clinical measurements, stratified by genetic burden. Liver involvement was highest in the liver-dominant group, showing a median LIS of 5.7 compared to 4.0 in the mixed/intermediate group and 4.1 in the lung-dominant group (*p* < 0.001), corresponding to a 40 to 45 percent higher liver disease burden. Pulmonary involvement scores demonstrated corresponding separation, with a median PIS of 8.0 for the lung-dominant group versus 2.0 and 1.0 for the mixed/intermediate and liver-dominant groups, respectively (*p* < 0.001). Direct-variable evaluation showed that the liver-dominant group had an elevated median liver stiffness of 6.4 kPa compared to 4.8 and 4.9 kPa in the other groups, representing a 30 to 35 percent increase. The lung-dominant group exhibited a reduced median FEV1 of 48.0 percent predicted versus 89.0 and 95.0 percent in the other groups (*p* < 0.001), corresponding to an absolute reduction of 40 to 50 percentage points. Genotype-stratified analyses revealed that these structural separations were preserved across all Z-allele burdens. Within the Z = 2 stratum, liver-dominant patients exhibited LIS values clustering in the 6.0 to 10.0 range and liver stiffness values extending between 20.0 and 60.0 kPa. Conversely, lung-dominant individuals within the Z = 2 stratum demonstrated FEV1 values clustering below 50.0 to 60.0 percent predicted, representing a 40 to 60 percentage point reduction relative to the mixed/intermediate and liver-dominant groups. The mixed/intermediate category occupied an intermediate position across both plots.

### 3.3. Geometric Mapping and Clinical Drivers of Disease Trajectories

Continuous hepatic and pulmonary phenotypic distributions were mapped into a ternary coordinate system to evaluate geometric disease trajectories. Subsequently, multivariable multinomial logistic regression modeling was applied to identify the independent clinical determinants driving organ-specific dominance.

**Figure 3:**
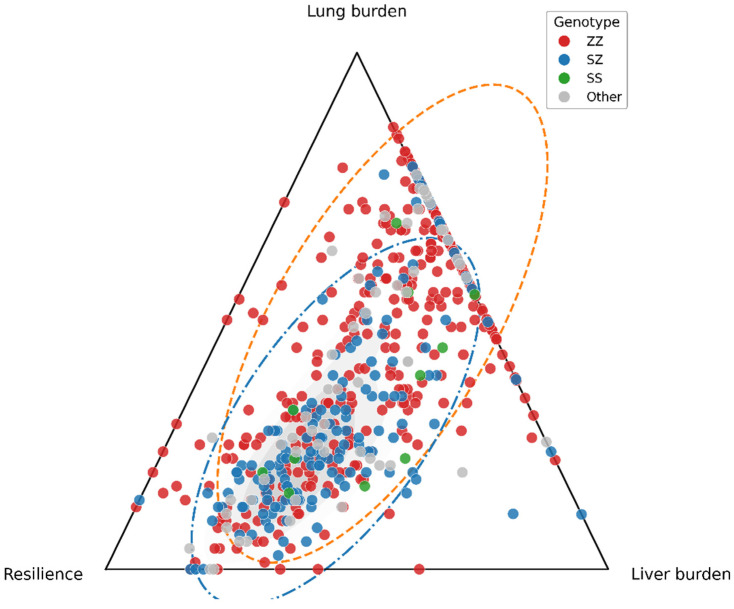
Global and Genotype-Stratified Ternary Mapping of Hepatic and Pulmonary Phenotypes. Note. Ternary density plot illustrating the distribution of patients across a continuous two-dimensional phenotypic space. The three axes represent relative pulmonary burden, relative hepatic burden, and low combined organ burden (resilience), derived from a geometric coordinate transformation of the normalized Pulmonary Involvement Score (PIS) and Liver Involvement Score (LIS). The distribution is stratified by genotype (ZZ, SZ, SS, and Other), with covariance ellipses representing data dispersion for the ZZ and SZ genotype groups.

Figure 3 illustrates the geometric distribution of patients across the hepatic and pulmonary phenotypic space. Individuals with more severe genetic mutations exhibit dispersion away from the low combined burden pole. For the ZZ genotype group, the distribution extended toward the extreme margins of both the lung-dominant and liver-dominant axes, diverging from the central coordinate space.

**Figure 4:**
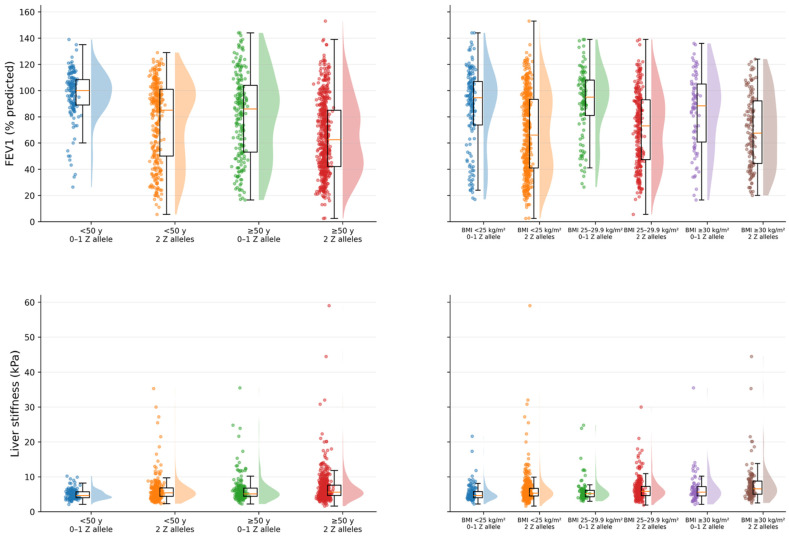
Distribution of Pulmonary and Hepatic Phenotypes Across Age, Body Mass Index, and Genotype Strata. Note. Raincloud plots illustrating the distribution of pulmonary function (forced expiratory volume in one second [FEV1], % predicted; top row) and liver stiffness (kPa; bottom row) across combined strata of age, body mass index (BMI), and *SERPINA1* genotype burden. The left-sided plots display distributions across age categories (<50 versus ≥50 years) stratified by genotype burden (0–1 versus 2 Z alleles). The right-sided plots display distributions across BMI categories (<25.0, 25.0–29.9, and ≥30.0 kg/m^2^) stratified by genotype. Each plot combines kernel density estimation (violin plot), boxplots (indicating the median and interquartile range), and individual observations. Abbreviations: FEV1, forced expiratory volume in one second; BMI, body mass index; kPa, kilopascals.

Figure 4 shows the distribution of FEV1 and liver stiffness across age, BMI, and genotype strata. FEV1 exhibited lower values in individuals carrying two Z alleles compared to those with 0–1 alleles (F = 376.6, *p* < 0.001, η^2^ = 0.098), accounting for 10.0% of the variance. Age was associated with reduced FEV1 (F = 241.4, *p* < 0.001, η^2^ = 0.065). BMI was not statistically associated with FEV1 (F = 2.23, *p* = 0.107, η^2^ = 0.001), showing overlapping distributions across BMI categories. Liver stiffness was associated with BMI, with higher BMI corresponding to increased stiffness values (F = 9.96, *p* < 0.001, η^2^ = 0.017). Genotype (F = 11.15, *p* < 0.001, η^2^ = 0.009) and age (F = 15.82, *p* < 0.001, η^2^ = 0.013) also exhibited associations with liver stiffness.

To identify the independent clinical and demographic determinants of these divergent trajectories, a multivariable multinomial logistic regression model was constructed using the mixed/intermediate phenotype as the reference category (Table 2).

Compared with the mixed/intermediate phenotype, liver-dominant classification was associated with a higher BMI (OR 1.03; 95% CI 1.00–1.07, *p* = 0.035), male sex (OR 1.66; 95% CI 1.20–2.28, *p* = 0.002), and never-smoking status compared with current smoking (OR 1.44; 95% CI 1.03–2.00, *p* = 0.032). Lung-dominant classification was associated with increasing age (OR 1.03; 95% CI 1.02–1.05, *p* < 0.001), lower BMI (OR 0.96; 95% CI 0.93–1.00, *p* = 0.030), and lower odds among never-smokers compared with current smokers (OR 0.36; 95% CI 0.25–0.50, *p* < 0.001). Individuals carrying one Z-allele had significantly lower odds of lung-dominant classification compared with those carrying two Z alleles (OR 0.34; 95% CI 0.23–0.51, *p* < 0.001). Alcohol exposure categories did not reach statistical significance. In the direct comparison between liver-dominant and lung-dominant phenotypes, a higher BMI (OR 1.09; 95% CI 1.03–1.14, *p* = 0.002), male sex (OR 1.85; 95% CI 1.15–2.99, *p* = 0.012), never-smoking status (OR 3.32; 95% CI 1.68–6.59, *p* < 0.001), and lower Z-allele burden were associated with liver-dominant classification. Conversely, increasing age was associated with a modest shift toward pulmonary predominance (OR 0.98; 95% CI 0.96–1.00, *p* = 0.034). Sensitivity analyses using alternative dominance thresholds (±0.5 SD and ±1.5 SD) yielded directionally consistent associations (Supplementary Table S3). In complementary continuous analyses, restricted cubic spline terms did not improve model fit when LIS was modeled as a function of PIS (Supplementary Table S5A) or when PIS was modeled as a function of LIS (Supplementary Table S5B). Similarly, multivariable continuous modeling of the dominance score showed that the clinical and genetic associations remained directionally consistent (Supplementary Table S5C).

## 4. Discussion

### 4.1. Principal Interpretation of the Findings

The primary finding of this international cohort study is that structural and functional impairments of the lung and liver in Alpha-1 Antitrypsin Deficiency (AATD) manifest as statistically independent clinical dimensions. While classical stratification by genetic burden confirms the expected overarching severity gradients across the cohort (Table S1), the isolation of patients by phenotypic dominance reveals distinct, non-overlapping clinical profiles that cannot be explained by the *SERPINA1* genotype alone (Table 1). Notably, the near-zero cross-domain correlations between respiratory and hepatic variables persisted after adjustment for shared demographic factors (Figure 1). Our results confirm this dissociation of lung and liver damage, demonstrating that they follow independent and uncorrelated trajectories. This independence is maintained despite the asymmetric clinical severity observed between advanced respiratory impairment and the earlier stages of hepatic fibrotic changes in this cohort.

### 4.2. Integration with Previous Literature

Previous clinical frameworks have frequently conceptualized AATD as a unified systemic proteinopathy, operating under the implicit premise that severe end-organ damage in one domain signals a generalized susceptibility to systemic deterioration [17]. Fragmented clinical observations have occasionally reported isolated organ manifestations, particularly within aging cohorts [18]. The present findings synthesize these isolated observations into a quantitatively validated framework. The preservation of structural separation across all genetic strata (Figure 2) indicates that, while the Z-allele dictates the basal architectural risk [19], it does not strictly govern the eventual dominance of hepatic or pulmonary impairment. This geometric divergence is visually corroborated by geometric mapping, wherein patients with advanced disease cluster at the extreme, orthogonal margins of the phenotypic space rather than converging toward combined cardiopulmonary and hepatic failure (Figure 3). These results extend and rigorously quantify recent evidence suggesting that liver and lung manifestations in AATD follow decoupled trajectories [20,21].

### 4.3. Clinical and Conceptual Implications

The statistical independence of the hepatic and pulmonary axes suggests a necessary refinement in clinical management and prognostic stratification [22,23]. Currently, the identification of severe emphysema often prompts clinical apprehension regarding concurrent advanced liver fibrosis, leading to generalized prognostic assumptions [24,25]. The present data indicate that systemic prognostication models based solely on genetic burden may not fully capture individualized risk [26,27]. Risk stratification should evolve toward multidimensional, organ-specific clinical evaluations [28]. Clinical monitoring algorithms must evaluate pulmonary and hepatic risks as independent entities, recognizing that the absence of severe disease in one organ provides no reliable statistical assurance regarding the structural integrity of the other [29].

### 4.4. Mechanistic or Interpretative Considerations

The divergent trajectories observed align with the fundamental pathobiological dichotomy inherent to AATD [30]. The pulmonary loss-of-function and hepatic gain-of-function mechanisms appear to be modulated by distinct environmental and metabolic factors rather than operating in tandem [31]. As demonstrated by the multivariable models, increasing age and a history of smoking were independently associated with lung-dominant phenotypes, consistent with the cumulative impact of oxidative stress exacerbating the protease-antiprotease imbalance [32]. Conversely, a higher body mass index was significantly associated with liver dominance, independent of the Z-allele burden. Given the uniform steatosis burden (CAP) across groups, this suggests that metabolic dysfunction may synergistically amplify the proteotoxic stress of mutant polymer accumulation via pathways distinct from isolated lipid accumulation, prioritizing hepatic fibrogenesis over pulmonary decline (Figure 4, Table 2). The observation that specific demographic and metabolic factors differentially associate with phenotypic expression supports the hypothesis of a highly modular pathobiology modulated by external insults [33]. At the molecular level, these divergent organ-specific trajectories may be mediated by epigenetic mechanisms, such as smoking-induced DNA methylation in the respiratory tract, alongside alterations in autophagy and histone modifications that influence the hepatic clearance of toxic, misfolded *SERPINA1* polymers [4,31].

### 4.5. Strengths and Methodological Value

A primary methodological strength of this investigation is the utilization of a large, deeply phenotyped international cohort, providing the statistical power necessary to evaluate independent cross-organ associations while controlling for critical covariates. The operationalization of disease severity through continuous composite clinical indices rather than binary diagnostic thresholds allowed for the precise geometric mapping of disease trajectories across a continuous severity spectrum [34]. The strict application of non-linear partial correlations and complete-case multivariable modeling ensures that the observed dissociation represents a verifiable clinical phenomenon rather than an artifact of statistical imputation or unmeasured shared demographic confounding.

### 4.6. Limitations

These findings must be interpreted in the context of specific methodological limitations. First, the cross-sectional design precludes the establishment of temporal causality or the evaluation of longitudinal within-patient transitions between phenotypic states. Consequently, survivor bias may influence the observed distributions, particularly in older individuals homozygous for the Z-allele, when extreme combined phenotypes could be underrepresented due to early mortality. Second, the strict complete-case analysis strategy utilized to preserve internal validity necessitated the exclusion of patients with partial data. Although included and excluded participants did not show major demographic differences, this requirement may introduce selection bias toward individuals managed in centers with more systematic multidisciplinary assessment and follow-up. Conversely, the excluded cohort may include less severely affected individuals who did not require advanced testing, such as DLCO measurement or liver elastography. This conservative approach was nevertheless essential to avoid the spurious covariance structures frequently generated by multiple imputation techniques in cross-domain correlation analyses. Third, although the LIS and PIS provide a standardized framework for multidimensional organ-specific phenotyping in this registry, they remain exploratory composite indices requiring external validation before clinical implementation. Future longitudinal studies should assess their reproducibility and prognostic performance. Fourth, hepatic phenotyping relied on non-invasive structural and biochemical markers rather than histological confirmation via liver biopsy. Transient elastography and the FIB-4 index are extensively validated, widely implemented prognostic tools that accurately reflect clinically meaningful fibrotic burden, making them appropriate and ethically justified for large-scale epidemiological mapping [35,36]. Fifth, estimates for alcohol exposure in the multinomial models should be interpreted cautiously because the wide confidence intervals likely reflect the small size of the non-exposure reference group.

### 4.7. Future Directions

Future longitudinal studies should determine whether patients move across the ternary phenotypic space over time, quantify organ-specific progression rates, and identify clinically meaningful transitions associated with subsequent pulmonary, hepatic, and survival outcomes. Such studies may clarify whether liver-dominant, lung-dominant, and mixed/intermediate phenotypes represent stable profiles or dynamic states that evolve with aging, metabolic burden, smoking exposure, and disease progression. Incorporating direct measures of abdominal obesity and visceral adiposity may further refine the characterization of metabolic contributions to organ-specific disease trajectories.

## 5. Conclusions

Hepatic and pulmonary disease severities in Alpha-1 Antitrypsin Deficiency operate as statistically independent clinical dimensions. While the *SERPINA1* genetic burden establishes the basal architectural susceptibility, divergence toward organ-specific phenotypic dominance is independently modulated by distinct environmental and metabolic factors. These findings demonstrate the inadequacy of unidimensional prognostic models based solely on genotype. Notably, smoking acts as a critical factor that shifts the clinical expression of AATD toward a pulmonary-dominant phenotype. Consequently, the clinical management of Alpha-1 Antitrypsin Deficiency must evolve toward multidimensional, organ-specific risk stratification algorithms to ensure accurate prognostication and targeted therapeutic monitoring.

## Figures and Tables

**Table 1:** Baseline Clinical Characteristics Stratified by Phenotypic Dominance Category.

Variable	Mixed/Intermediate (*n* = 258)	Liver-Dominant (*n* = 476)	Lung-Dominant (*n* = 483)	*p*-Value
**Demographics**				
Age, years	51.5 [39.8, 63.0]	54.0 [41.0, 64.0]	57.0 [50.0, 66.0]	<0.001
BMI, kg/m^2^	24.9 [22.6, 28.0]	25.8 [23.2, 29.6]	24.4 [22.2, 27.4]	<0.001
Male sex, *n* (%)	118 (45.7%)	273 (57.4%)	257 (53.2%)	0.011
**Comorbidities**				
Diabetes severity, *n* (%)				0.050
Without complications	14 (5.4%)	36 (7.6%)	21 (4.3%)	
With complications	0 (0.0%)	5 (1.1%)	2 (0.4%)	
Hypertension (Yes), *n* (%)	44 (17.1%)	103 (21.6%)	100 (20.7%)	0.324
Metabolic burden (MFB), *n* (%)				0.005
Low	64 (25.4%)	91 (19.6%)	132 (27.8%)	
Intermediate	70 (27.8%)	117 (25.2%)	136 (28.6%)	
High	118 (46.8%)	257 (55.3%)	207 (43.6%)	
**Exposure/Genotype**				
Smoking status, *n* (%)				<0.001
Ex-smokers	110 (42.6%)	179 (37.6%)	326 (67.5%)	
Never-smokers	131 (50.8%)	272 (57.1%)	134 (27.7%)	
Smokers	13 (5.0%)	25 (5.3%)	22 (4.6%)	
Z-allele count, *n* (%)				<0.001
0	8 (3.1%)	28 (5.9%)	19 (4.0%)	
1	85 (32.9%)	141 (29.9%)	69 (14.5%)	
2	165 (64.0%)	302 (64.1%)	389 (81.6%)	
**Pulmonary Function**				
FEV1% predicted	89.0 [68.0, 103.0]	95.0 [80.0, 108.0]	48.0 [35.0, 69.0]	<0.001
FVC % predicted	101.5 [87.0, 112.0]	100.0 [90.0, 110.0]	91.6 [73.8, 109.0]	<0.001
FEV1/FVC	0.73 [0.56, 0.80]	0.77 [0.67, 0.83]	0.44 [0.34, 0.55]	<0.001
DLCO % predicted	79 [59.0–97.0]	85 [67.0–97.0]	51.75 [38–64.25]	<0.001
Pulmonary Involvement Score (PIS)	2.0 [1.0, 6.0]	1.0 [0.0, 3.0]	8.0 [6.0, 9.0]	<0.001
**Hepatic Function**				
ALT, U/L	25.0 [18.0, 36.0]	30.0 [21.0, 48.0]	23.0 [17.9, 33.0]	<0.001
AST, U/L	25.0 [19.0, 32.2]	29.0 [22.0, 40.3]	24.0 [20.0, 30.0]	<0.001
GGT, U/L	24.0 [16.0, 43.3]	34.0 [20.0, 59.3]	28.0 [20.0, 44.0]	<0.001
FIB-4	0.97 [0.62, 1.40]	1.30 [0.85, 2.20]	1.06 [0.81, 1.36]	0.002
Liver stiffness, kPa	4.8 [4.0, 6.3]	6.4 [5.3, 8.6]	4.9 [4.0, 6.0]	<0.001
CAP, dB/m	239.5 [205.0, 289.5]	252.5 [213.8, 299.3]	255.0 [218.0, 295.0]	0.115
Liver Involvement Score (LIS)	4.00 [3.29, 5.70]	5.72 [4.66, 7.74]	4.06 [3.29, 5.03]	<0.001

Note. Data are presented as medians [interquartile ranges] for continuous variables and absolute counts (percentages) for categorical variables. Between-group comparisons were evaluated using the Kruskal–Wallis test for continuous variables and chi-square tests for categorical variables. Phenotypic dominance categories were defined using the standardized difference between the Liver Involvement Score (LIS) and the Pulmonary Involvement Score (PIS), calculated as Z(LIS) − Z(PIS). Patients were classified as liver-dominant if the score exceeded +1.0 standard deviation, lung-dominant if the score was below −1.0 standard deviation, and mixed/intermediate otherwise. The LIS is a 0–10 composite severity index integrating structural (liver stiffness, 0–5 points), biochemical (FIB-4 index, 0–3 points), and clinical (documented cirrhosis/hepatocellular carcinoma, 0–2 points) parameters. The PIS is a 0–10 composite index representing pulmonary disease burden. Metabolic burden (MFB) is a composite clinical score; the detailed methodology for its calculation and stratification is provided in Section 2.3. Abbreviations: BMI, body mass index; FEV1, forced expiratory volume in one second; FVC, forced vital capacity; DLCO, diffusing capacity of the lungs for carbon monoxide; ALT, alanine aminotransferase; AST, aspartate aminotransferase; GGT, gamma-glutamyl transferase; FIB-4, Fibrosis-4 index; CAP, controlled attenuation parameter; MFB, metabolic burden.

**Table 2:** Multinomial Logistic Regression for Phenotypic Dominance Category.

Predictor	Liver-Dominant vs. Mixed/Intermediate OR [95% CI]	*p*-Value	Lung-Dominant vs. Mixed/Intermediate OR [95% CI]	*p*-Value	Liver-Dominant vs. Lung-Dominant OR [95% CI]	*p*-Value
Age, per year	1.01 [1.00, 1.02]	0.117	1.03 [1.02, 1.05]	<0.001	0.98 [0.96–1.00]	0.034
BMI, per kg/m^2^	1.03 [1.00, 1.07]	0.035	0.96 [0.93, 1.00]	0.030	1.09 [1.03–1.14]	0.002
Male sex (Ref: Female sex)	1.66 [1.20–2.28]	0.002	1.21 [0.87–1.68]	0.258	1.85 [1.15–2.99]	0.012
Smoking status: Ex-smokers (Ref: Smokers)	0.81 [0.38, 1.73]	0.587	0.99 [0.45, 2.20]	0.987	0.54 [0.18–1.62]	0.272
Smoking status: Never-smokers (Ref: Smokers)	1.44 [1.03–2.00]	0.032	0.36 [0.25–0.50]	<0.001	3.32 [1.68–6.59]	<0.001
Alcohol exposure: Hazardous (Ref: None)	1.31 [0.10, 16.79]	0.836	1.07 [0.08, 14.75]	0.957	1.24 [0.07–22.16]	0.883
Alcohol exposure: Low-moderate (Ref: None)	2.70 [0.23, 31.27]	0.428	1.61 [0.13, 20.50]	0.712	0.83 [0.04–16.16]	0.901
Z-allele count: 0 (Ref: 2 alleles)	2.22 [0.94, 5.27]	0.070	1.15 [0.46, 2.88]	0.058	4.14 [1.22–14.10]	0.023
Z-allele count: 1 (Ref: 2 alleles)	0.95 [0.67, 1.34]	0.758	0.34 [0.23, 0.51]	<0.001	3.62 [1.96–6.69]	<0.001

Note. Multinomial logistic regression evaluating clinical predictors of phenotypic dominance. The mixed/intermediate category serves as the reference outcome for the liver-dominant and lung-dominant comparisons. The liver-dominant versus lung-dominant contrast represents the direct comparison between the two extreme phenotypic categories. Phenotypic dominance categories were defined using the standardized difference between the Liver Involvement Score and the Pulmonary Involvement Score, calculated as Z(LIS) − Z(PIS). Patients were classified as liver-dominant if the score was >+1.0 standard deviation (SD), lung-dominant if the score was <−1.0 SD, and mixed/intermediate if the score fell between −1.0 and +1.0 SD. Odds ratios (ORs) greater than 1.00 indicate increased relative odds of classification into the specified dominance category. The model adjusts simultaneously for age, body mass index (BMI), sex, smoking status, alcohol exposure, and SERPINA1 Z-allele count. Abbreviations: OR, odds ratio; CI, confidence interval; BMI, body mass index; SD, standard deviation.

## Data Availability

Data availability on request from the corresponding author.

## Supplementary Materials

The following supporting information can be downloaded at https://www.mdpi.com/article/10.3390/biom16070940/s1, Supplementary Methods: Detailed Scoring Formulas for Composite Indices; Supplementary Table S1: Complete dataset vs. participants with missing dominance score; Supplementary Table S2: Agreement between alternative phenotypic dominance definitions (Cohen’s κ); Supplementary Table S3: Sensitivity analysis: Multinomial regression across alternative phenotypic dominance definitions; Supplementary Table S4: Collinearity diagnostics for multinomial regression predictors; Supplementary Table S5A: Restricted cubic spline model predicting Liver Involvement Score (LIS); Supplementary Table S5B: Restricted cubic spline model predicting Pulmonary Involvement Score (PIS); Supplementary Table S5C: Restricted cubic spline model predicting continuous dominance score; Supplementary Figure S1: Distribution of phenotypic dominance across body mass index (BMI) categories.

## Abbreviations

The following abbreviations are used in this manuscript:

AATD	Alpha-1 Antitrypsin Deficiency
EARCO	European Alpha-1 Research Collaboration
FEV1	Forced Expiratory Volume in one second
FVC	Forced Vital Capacity
DLCO	Diffusing Capacity of the Lungs for Carbon Monoxide
LSM	Liver Stiffness Measurement
CAP	Controlled Attenuation Parameter
ALT	Alanine Aminotransferase
AST	Aspartate Aminotransferase
GGT	Gamma-Glutamyl Transferase
FIB-4	Fibrosis-4 Index
LIS	Liver Involvement Score
PIS	Pulmonary Involvement Score
BMI	Body Mass Index
MFB	Metabolic Burden Score
SD	Standard Deviation
OR	Odds Ratio
CI	Confidence Interval
ERS	European Respiratory Society
ERN-LUNG	European Reference Network LUNG
