# Supplementary material for: Dissociation of the Hepatic and Pulmonary Axes in Alpha-1 Antitrypsin Deficiency: Independent Trajectories of Organ-Specific Disease
Source: Biomolecules. 2026 Jun 24;16(7):940. doi: 10.3390/biom16070940 (PMC13407244; doi:10.3390/biom16070940)
Supplement: Supplementary file 1 [file biomolecules-16-00940-s001.zip › biomolecules-4322088-supplementary.pdf]

Supplementary Methods: Detailed Scoring Formulas for Composite Indices

Pulmonary Involvement Score (PIS)

The PIS was calculated as a domain-based continuous index integrating functional and structural pulmonary impairment. Each available domain was scored continuously across predefined severity ranges, and the final score was prorated to a 0–10 scale according to the maximum possible score for the domains with available data. The PIS was calculated only when at least three of the four pulmonary domains were available.

Airflow obstruction domain:

$$PIS\_obstr = \min(4, \max(0, [(0.70 - FEV1/FVC) / 0.20] \times 4))$$

FEV1 impairment domain:

$$PIS\_FEV1 = \min(4, \max(0, [(80 - FEV1\ \%\ predicted) / 45] \times 4))$$

DLCO impairment domain:

$$PIS\_DLCO = \min(4, \max(0, [(80 - DLCO\ \%\ predicted) / 45] \times 4))$$

Structural lung damage domain:

PIS_struct = 2 points if emphysema and/or bronchiectasis was present, and 0 points otherwise.

The raw pulmonary score was calculated as:

$$PIS\_raw = PIS\_obstr + PIS\_FEV1 + PIS\_DLCO + PIS\_struct$$

The maximum possible observed score was calculated as the sum of the maximum scores for the domains available in each participant. The final prorated score was calculated as:

$$PIS = (PIS\_raw / PIS\_max\_observed) \times 10$$

Liver Involvement Score (LIS)

The LIS was calculated using a similar domain-based continuous and prorated structure, integrating liver stiffness, fibrosis biomarker burden, and clinically documented liver disease. The LIS was calculated only when at least two of the three hepatic domains were available.

Liver stiffness domain:

$$LIS\_LSM = \min(5, \max(0, [\text{liver stiffness measurement (kPa)} / 9.5] \times 5))$$

Fibrosis biomarker domain:

$$LIS\_FIB4 = \min(3, \max(0, [FIB\text{-}4 / 3.25] \times 3))$$

Clinical liver disease domain:

LIS_clin = 2 points if cirrhosis or hepatocellular carcinoma was present, 1 point if ascites was present in the absence of cirrhosis or hepatocellular carcinoma, and 0 points otherwise.

The raw liver score was calculated as:

$$LIS\_raw = LIS\_LSM + LIS\_FIB4 + LIS\_clin$$

The maximum possible observed score was calculated as the sum of the maximum scores for the domains available in each participant. The final prorated score was calculated as:

LIS = (LIS_raw / LIS_max_observed) × 10

Supplementary Table S1. Complete dataset vs. participants with missing dominance score.

Variable	Missing dominance score (Excluded n = 2662)	Included data (N = 1217)	p-value
Age (years)	54.5 ± 22.2	54.8 ± 25.1	0.620
Sex			0.560
• Female	48.4%	49.2%	
• Male	51.6%	50.8%	
BMI (kg/m²)	26.0 ± 9.0	26.1 ± 10.5	0.780
Smoking status			0.172
• Never smoker	41.0%	39.4%	
• Ex-smoker	51.7%	52.2%	
• Smoker	7.3%	8.4%	
Alcohol exposure			0.304
• No alcohol	2.8%	4.2%	
• Low–moderate	86.1%	85.1%	
• Hazardous	11.1%	10.7%	
FEV1 (% predicted)	75.7 ± 31.2	76.4 ± 31.7	0.425
DLCO	6.8 ± 3.7	7.0 ± 4.0	0.128
Pulmonary Involvement Score (PIS)	4.3 ± 3.6	4.2 ± 3.6	0.385
Liver stiffness (kPa)	6.3 ± 3.9	5.6 ± 2.8	0.150
FIB-4	1.4 ± 2.5	1.3 ± 1.4	0.119
CAP (dB/m)	251.6 ± 66.5	246.1 ± 80.6	0.763
Liver Involvement Score (LIS)	5.1 ± 1.9	4.5 ± 1.8	0.089

Note. Data are presented as mean ± standard deviation or percentages. Abbreviations: BMI, body mass index; FEV1, forced expiratory volume in one second; DLCO, diffusing capacity of the lungs for carbon monoxide; PIS, Pulmonary Involvement Score; FIB-4, Fibrosis-4 index; CAP, controlled attenuation parameter; LIS, Liver Involvement Score.

There were no statistically significant differences between the full dataset and participants with missing dominance scores across demographic, pulmonary, and hepatic variables, including DLCO, pulmonary involvement score (PIS), liver stiffness, FIB-4, CAP, and liver involvement score (all $p > 0.05$). A non-significant trend toward lower liver involvement scores in participants with missing dominance data was observed ($p = 0.089$). Similarly, smoking status and alcohol exposure distributions did not differ significantly.

Supplementary Table S2. Agreement between alternative phenotypic dominance definitions

Comparison	Agreement (%)	κ (Kappa)	SE	Z	p-value
±0.5 SD vs ±1.0 SD	93.4	0.900	0.013	71.95	<0.001
±1.0 SD vs ±1.5 SD	95.2	0.924	0.011	84.10	<0.001

Note. To evaluate the robustness of phenotypic dominance classification, alternative threshold definitions were applied based on standardized differences between liver and pulmonary involvement. The primary definition used ±1.0 SD thresholds, with liver-dominant classification assigned to values > +1.0 SD and lung-dominant classification assigned to values < −1.0 SD. A more inclusive definition used ±0.5 SD thresholds, whereas a more stringent definition used ±1.5 SD thresholds; values within each threshold range were categorized as mixed/intermediate phenotype.

Agreement between alternative phenotypic dominance definitions was high. The comparison between the ±0.5 SD and primary ±1.0 SD definitions showed 93.4% agreement with near-perfect concordance by Cohen's κ (κ = 0.900, SE = 0.013, $p < 0.001$). Similarly, the comparison between the primary ±1.0 SD and more stringent ±1.5 SD definitions showed 95.2% agreement (κ = 0.924, SE = 0.011, $p < 0.001$). These findings indicate that the phenotypic dominance classification was stable across alternative distribution-based thresholds.

Supplementary Table S3. Sensitivity analysis: multinomial regression across alternative phenotypic dominance definitions.

Comparison	Predictor	±0.5 SD threshold OR (95% CI), p	±1.0 SD threshold OR (95% CI), p	±1.5 SD threshold OR (95% CI), p
Liver-dominant vs Mixed/intermediate	Age	1.01 (1.00–1.02), p = 0.134	1.01 (1.00–1.02), p = 0.117	1.01 (1.00–1.02), p = 0.130
Liver-dominant vs Mixed/intermediate	BMI	1.05 (1.02–1.08), p = 0.002	1.03 (1.00–1.07), p = 0.035	1.04 (1.00–1.07), p = 0.043
Liver-dominant vs Mixed/intermediate	Current smoker	1.13 (0.56–2.28), p = 0.732	1.25 (0.59–2.66), p = 0.559	0.89 (0.42–1.91), p = 0.771
Liver-dominant vs Mixed/intermediate	Male sex	1.58 (1.17–2.14), p = 0.003	1.66 (1.20–2.28), p = 0.002	1.81 (1.28–2.55), p < 0.001
Liver-dominant vs Mixed/intermediate	Never smoker	1.42 (1.04–1.95), p = 0.027	1.44 (1.03–2.00), p = 0.032	1.62 (1.13–2.32), p = 0.009
Liver-dominant vs Mixed/intermediate	Z-count = 2	1.09 (0.80–1.50), p = 0.579	0.95 (0.68–1.33), p = 0.766	0.87 (0.61–1.25), p = 0.449
Lung-dominant vs Mixed/intermediate	Age	1.03 (1.02–1.04), p < 0.001	1.03 (1.02–1.05), p < 0.001	1.04 (1.02–1.05), p < 0.001
Lung-dominant vs Mixed/intermediate	BMI	0.96 (0.93–1.00), p = 0.032	0.96 (0.93–0.99), p = 0.030	0.96 (0.93–1.00), p = 0.035
Lung-dominant vs Mixed/intermediate	Current smoker	0.88 (0.42–1.83), p = 0.731	1.03 (0.47–2.27), p = 0.938	0.80 (0.36–1.76), p = 0.572
Lung-dominant vs Mixed/intermediate	Male sex	1.09 (0.80–1.49), p = 0.571	1.21 (0.87–1.68), p = 0.258	1.37 (0.96–1.96), p = 0.079
Lung-dominant vs Mixed/intermediate	Never smoker	0.32 (0.23–0.45), p < 0.001	0.36 (0.25–0.50), p < 0.001	0.44 (0.30–0.64), p < 0.001
Lung-dominant vs Mixed/intermediate	Z-count = 2	2.74 (1.92–3.91), p < 0.001	2.49 (1.71–3.61), p < 0.001	2.31 (1.56–3.44), p < 0.001

Note. Reference outcome category = Mixed/intermediate. Models included age, sex, smoking status, body mass index, and Z-count. Variables directly contributing to phenotypic scores (FEV1 and liver stiffness) were excluded to maintain statistical independence. Intercepts are omitted for readability. OR, odds ratio; CI, confidence interval; SD, standard deviation.

To evaluate the robustness of the primary classification schema, sensitivity analyses utilizing alternative phenotypic dominance definitions (±0.5 SD, ±1.0 SD, and ±1.5 SD thresholds) were performed using multivariable multinomial logistic regression. Effect sizes and statistical significance remained directionally consistent across all threshold specifications.

Supplementary Table S4. Collinearity diagnostics for multinomial regression predictors.

Predictor	Tolerance	VIF
Age	0.96	1.04
BMI	0.96	1.04
Z allele count	0.95	1.05
Sex (female reference)	0.97	1.03
Smoking status (Never/Ex vs ref)	0.09	1.06
Smoking status (Current vs ref)	0.09	1.17
Alcohol exposure (Low–moderate)	0.92	1.09
Alcohol exposure (None)	0.91	1.09

Note. Abbreviations: VIF, variance inflation factor; BMI, body mass index.

Collinearity diagnostics showed low VIF values (< 2.5) for all predictors. There was no evidence of problematic collinearity among continuous predictors (all VIF ≈ 1.0).

Supplementary Table S5A. Restricted cubic spline model predicting Liver Involvement Score (LIS).

Predictor	B	SE	t	p	95% CI
Intercept	0.913	0.348	2.622	0.009	0.230 to 1.596
PIS	-0.078	0.055	-1.428	0.154	-0.186 to 0.029
PIS spline term	0.069	0.067	1.025	0.306	-0.063 to 0.201
Age	0.044	0.004	10.825	< 0.001	0.036 to 0.052
Male sex	0.955	0.103	9.254	< 0.001	0.752 to 1.157
Never smoker	-0.003	0.116	-0.022	0.982	-0.229 to 0.224
Current smoker	-0.112	0.242	-0.465	0.642	-0.588 to 0.363
Z-count = 2	0.276	0.119	2.321	0.020	0.043 to 0.510
BMI	0.049	0.010	4.763	< 0.001	0.029 to 0.070

Note. Knots for PIS were placed at the 10th, 50th, and 90th percentiles of the distribution (0, 4, and 9). Model summary: $R^2 = 0.185$, adjusted $R^2 = 0.179$, $F(8, 1146) = 32.5$, $p < 0.001$. Spline improvement over linear model: $F(1, 1146) = 1.05$, $p = 0.306$. SE, standard error; CI, confidence interval; BMI, body mass index.

To assess potential non-linear relationships without relying on categorical cutoffs, restricted cubic spline models were fitted to evaluate the continuous association between total pulmonary burden and hepatic severity. Formal testing did not identify evidence of a statistically significant non-linear association beyond the corresponding linear specification.

Supplementary Table S5B. Restricted cubic spline model predicting Pulmonary Involvement Score (PIS).

Predictor	B	SE	t	p	95% CI
Intercept	0.872	0.712	1.224	0.221	-0.525 to 2.269
LIS	-0.072	0.125	-0.573	0.567	-0.318 to 0.174
LIS spline term	0.010	0.177	0.057	0.954	-0.337 to 0.358
Age	0.089	0.006	15.029	< 0.001	0.077 to 0.101
Male sex	1.199	0.168	7.147	< 0.001	0.870 to 1.529
Never smoker	-2.765	0.167	-16.602	< 0.001	-3.092 to -2.438
Current smoker	-0.873	0.388	-2.252	0.024	-1.633 to -0.113
Z-count = 2	2.092	0.180	11.653	< 0.001	1.740 to 2.444
BMI	-0.066	0.017	-3.980	< 0.001	-0.099 to -0.034

Note. Knots for LIS were placed at the 10th, 50th, and 90th percentiles of the distribution (3.03, 4.72, and 7.82). Model summary: $R^2 = 0.425$, adjusted $R^2 = 0.421$, $F(8, 1146) = 106.0$, $p < 0.001$. Spline improvement over linear model: $F(1, 1146) = 0.00$, $p = 0.954$. SE, standard error; CI, confidence interval; BMI, body mass index.

A reciprocal multivariable continuous model was implemented using the Liver Involvement Score as a restricted cubic spline predictor to evaluate its continuous association with total pulmonary burden. Formal testing did not identify evidence of a statistically significant non-linear association between continuous hepatic metrics and overall respiratory burden.

Supplementary Table S5C. Restricted cubic spline model predicting the continuous dominance score

Predictor	B	SE	t	p	95% CI
Intercept	-0.616	0.297	-2.073	0.038	-1.199 to -0.033
Age	-0.019	0.005	-3.627	< 0.001	-0.029 to -0.009
Age spline term	0.022	0.006	3.578	< 0.001	0.010 to 0.034
Male sex	0.166	0.072	2.325	0.020	0.026 to 0.307
Never smoker	0.762	0.075	10.145	< 0.001	0.615 to 0.910
Current smoker	0.143	0.172	0.829	0.407	-0.195 to 0.480
Z-count = 2	-0.461	0.079	-5.807	< 0.001	-0.617 to -0.305
BMI	0.049	0.007	6.726	< 0.001	0.035 to 0.064

Note. The continuous dominance score was defined as $D = Z(LIS) - Z(PIS)$, with positive values indicating relatively greater liver involvement and negative values indicating relatively greater pulmonary involvement. Restricted cubic splines for age used knots at 33, 55, and 72 years. Spline improvement over the linear age model: $F(1, 1147) = 12.80$, $p < 0.001$.

To reduce reliance on categorical phenotype thresholds, the continuous dominance score was modeled as Z(LIS) − Z(PIS), with positive values indicating relatively greater liver involvement and negative values indicating relatively greater pulmonary involvement. Higher BMI, male sex, and never-smoking status were associated with higher dominance score values, whereas Z-count = 2 was associated with lower dominance score values. Age showed a nonlinear association with the continuous dominance score.

Supplementary Figure S1. Distribution of phenotypic dominance across body mass index (BMI) categories.

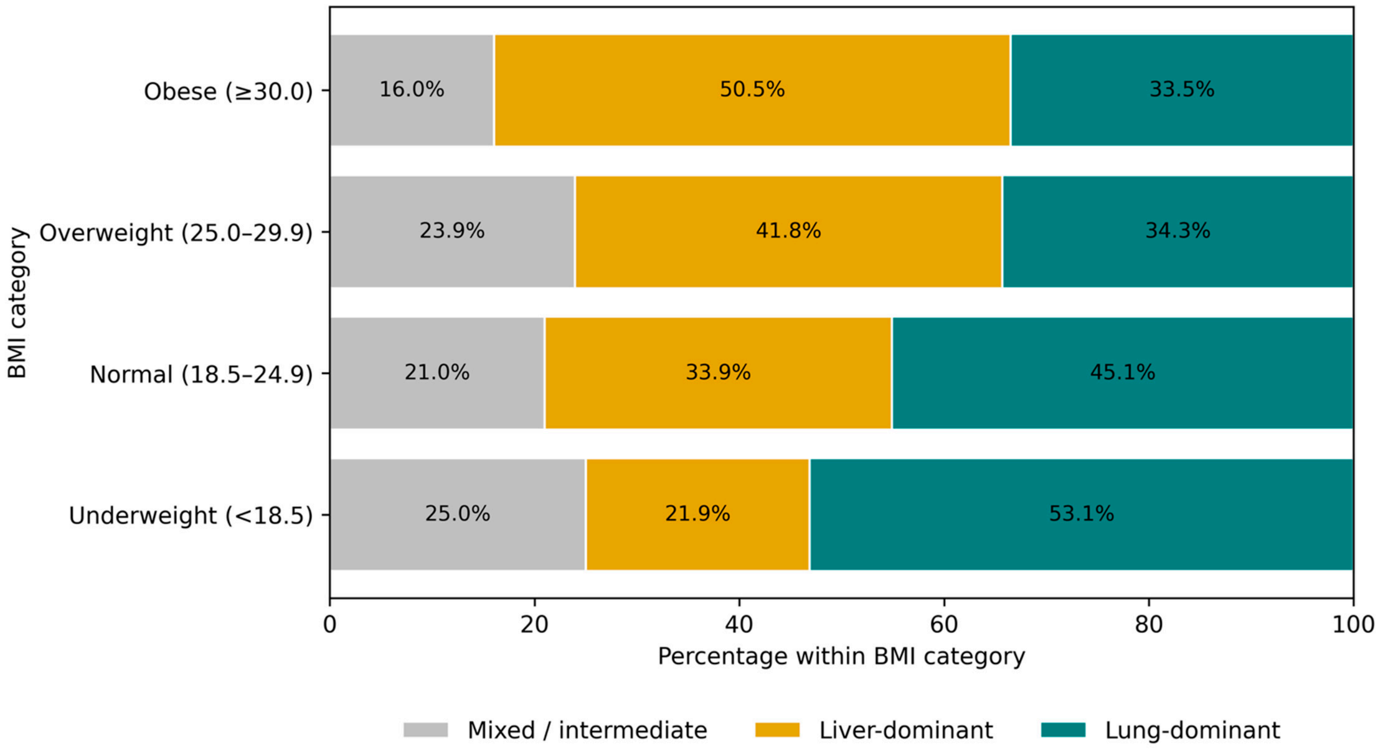

Note. The proportion of liver-dominant phenotypes increased progressively from 33.9% in normal-weight individuals to 50.5% in obese individuals. Conversely, the proportion of lung-dominant phenotypes decreased from 45.1% to 33.5% across the same BMI categories ($\chi^2[6] = 28.74$, $p < 0.001$).

Visual mapping of phenotypic distribution across standardized BMI categories supports a progressive association between increasing body mass and liver-dominant clinical presentations.
